# SymPortal: A novel analytical framework and platform for coral algal symbiont next‐generation sequencing *ITS2* profiling

**DOI:** 10.1111/1755-0998.13004

**Published:** 2019-04-26

**Authors:** Benjamin C. C. Hume, Edward G. Smith, Maren Ziegler, Hugh J. M. Warrington, John A. Burt, Todd C. LaJeunesse, Joerg Wiedenmann, Christian R. Voolstra

**Affiliations:** ^1^ Division of Biological and Environmental Science and Engineering (BESE), Red Sea Research Center King Abdullah University of Science and Technology (KAUST) Thuwal Saudi Arabia; ^2^ Center for Genomics and Systems Biology New York University Abu Dhabi Abu Dhabi UAE; ^3^ Faculty of Computer Science and Technology University of Cambridge UK; ^4^ Department of Biology The Pennsylvania State University University Park Pennsylvania; ^5^ Coral Reef Laboratory, Ocean and Earth Sciences University of Southampton Southampton UK; ^6^ Institute for Life Sciences University of Southampton Southampton UK

**Keywords:** ITS2, molecular ecology, multicopy, phylogentics, Symbiodiniaceae, SymPortal

## Abstract

We present SymPortal (SymPortal.org), a novel analytical framework and platform for genetically resolving the algal symbionts of reef corals using next‐generation sequencing (NGS) data of the *ITS2* rDNA. Although the *ITS2* marker is widely used to genetically characterize taxa within the family Symbiodiniaceae (formerly the genus *Symbiodinium)*, the multicopy nature of the marker complicates its use. Commonly, the intragenomic diversity resultant from this multicopy nature is collapsed by analytical approaches, thereby focusing on only the most abundant sequences. In contrast, SymPortal employs logic to identify within‐sample informative intragenomic sequences, which we have termed ‘defining intragenomic variants' (DIVs), to identify *ITS2*‐type profiles representative of putative Symbiodiniaceae taxa. By making use of this intragenomic *ITS2* diversity, SymPortal is able to resolve genetic delineations using the *ITS2* marker at a level that was previously only possible by using additional genetic markers. We demonstrate this by comparing this novel approach to the most commonly used alternative approach for NGS *ITS2* data, the 97% similarity clustering to operational taxonomic units (OTUs). The SymPortal platform accepts NGS raw sequencing data as input to provide an easy‐to‐use, standardization‐enforced, and community‐driven framework that integrates with a database to gain resolving power with increased use. We consider that SymPortal, in conjunction with ongoing large‐scale sampling and sequencing efforts, should play an instrumental role in making future sampling efforts more comparable and in maximizing their efficacy in working towards the classification of the global Symbiodiniaceae diversity.

## 1 INTRODUCTION

Corals and the ecosystems they construct have been among the most susceptible to climate change, but corals have been shown to exhibit variation in their responses to stress (Hughes et al., 2018; Norstrom et al., 2016). One important factor that contributes to reef resilience is the genetic identity of the algal symbionts harboured by the reef‐building corals (Hume et al., 2016; Kemp, Hernandez‐Pech, Iglesias‐Prieto, Fitt, & Schmidt, 2014; LaJeunesse et al., 2010; Rowan, 2004; Rowan, Knowlton, Baker, & Jara, 1997; Silverstein, Cunning, & Baker, 2015; Thornhill, Howells, Wham, Steury, & Santos, 2017). Effective characterization of these algal symbionts, family Symbiodiniaceae (formerly genus *Symbiodinium* containing Clades A–I, now equivalent to family Symbiodiniaceae, currently with seven named genera; LaJeunesse et al., 2018), is therefore important.

Despite the multitude of markers available for assessing Symbiodiniaceae diversity, the internal transcribed spacer 2 (*ITS2*) of the rRNA gene shows an uninterrupted popularity and remains the most commonly used marker (Cunning, Gates, & Edmunds, 2017; Fujise et al., 2018; Pochon, Putnam, & Gates, 2014; Smith, Ketchum, & Burt, 2017b; Varasteh, Shokri, Rajabi‐Maham, Behzadi, & Hume, 2018). However, this marker is multicopy in nature, which complicates its use. A single Symbiodiniaceae cell usually contains hundreds to thousands of rRNA gene copies (Arif et al., 2014; LaJeunesse, 2002; Thornhill, Lajeunesse, & Santos, 2007). On the one hand, diversity of *ITS2* sequences can come from sequence variations among these gene copies, giving rise to intragenomic diversity. On the other hand, sample sequence diversity may also be due to hosts associating with multiple genotypes of Symbiodiniaceae, referred to as intergenomic diversity (Sampayo, Dove, & Lajeunesse, 2009). Without the use of additional genetic markers, differentiating among these sources of variation can be challenging (Thornhill et al., 2007).

Historically, analyses have primarily used one of two techniques to characterize *ITS2* sequence diversity from PCR amplicons: denaturing gradient gel electrophoresis (DGGE) or molecular cloning‐and‐sequencing. Regardless of the technique used, the responsibility of characterizing different sequences as being due to intra‐ or intergenomic sources of variance lays with the researcher. Most analyses successfully incorporated intragenomic theory, leading to the core understanding that specific combinations of intragenomic sequences (commonly known as *ITS2* profiles) could differentiate, and therefore define, taxa (often referred to as so‐called ‘types’; LaJeunesse, 2002). An example is the identification of both the D1 and D4 sequences to characterize *Durusdinium trenchii*, formerly *Symbiodinium trenchii* (LaJeunesse, Wham, & Pettay, 2014). However, analyses interpreting every sequence as representative of a distinct Symbiodiniaceae genotype leads to erroneous inflation of Symbiodiniaceae diversity estimates (Apprill & Gates, 2007; Thornhill et al., 2007).

Whilst DGGE and to a lesser extent molecular cloning‐and‐sequencing approaches are still used to analyse the state of rDNA variation (Hume, D’Angelo, Burt, & Wiedenmann, 2018; Smith, Hume, Delaney, Wiedenmann, & Burt, 2017a; Varasteh et al., 2018; Wham, Ning, & LaJeunesse, 2017), contemporary analyses are starkly shifting towards employing next‐generation sequencing (NGS) technologies (Cunning et al., 2017; Hume et al., 2016; Hume, Ziegler et al., 2018; Smith, Ketchum et al., 2017b; Ziegler et al., 2017). These NGS approaches afford a greater sequencing depth, but yield sequence diversities orders of magnitude larger than previous methodologies. NGS approaches also overcome limitations associated with the gel‐based DGGE methodologies, such as, but not limited to, poor resolution between sequences and a sequence detection limit reliant on the effectiveness of the staining technique employed.

Despite the advances offered by NGS approaches, there is no consensus on how best to exploit their increased sequencing depth and differentiate between intragenomic and intergenomic sequence diversity. Initial analyses have used operational taxonomic unit (OTU) approaches to collapse sequence diversity at a 97% similarity threshold (Arif et al., 2014; Cunning et al., 2017). Yet other approaches have combined OTU analyses with searches for key *ITS2* type‐defining sequences in attempts to better exploit the data (Ziegler et al., 2017). Most recently, a minimum entropy decomposition (MED)‐based approach (referred to as the metahaplotype approach) has been used to consolidate the high diversity to a smaller set of core sequence nodes, based on biologically informative sequence positions rather than the fixed similarity thresholds of the OTU approach (Eren et al., 2015; Smith, Ketchum et al., 2017b).

However, these above‐mentioned approaches are ultimately limited in their ability to resolve taxa from NGS *ITS2* data. As numerous Symbiodiniaceae species share the same most abundant *ITS2* sequence, and a 1‐bp difference in this most abundant sequence may relate to evolutionary divergences of >10 million years (Thornhill, Lewis, Wham, & LaJeunesse, 2014), the fixed similarity threshold clustering of OTU approaches greatly limits their ability to maintain informative intragenomic structure and thus, resolve Symbiodiniaceae taxa. In contrast, the metahaplotype approach (Smith, Ketchum et al., 2017b) does retain intragenomic diversity information. However, it does not differentiate between intra‐ and intergenomic diversity and is thus limited to scenarios where samples only contain a single symbiont taxon (Smith, Ketchum et al., 2017b). To verify this single symbiont assumption, the method is reliant on an additional less conserved marker, the chloroplastic *psbA* noncoding region (*psbA* ^ncr^), a marker often used to differentiate between closely related Symbiodiniaceae taxa (LaJeunesse & Thornhill, 2011). While this marker may appear to be an attractive alternative to the *ITS2* region due to its power to resolve closely related taxa, its highly variable nature severely limits the taxonomic range over which returned sequences can be aligned, and therefore it is effective only for reconstructing fine‐level phylogenies among closely related taxa (Thornhill et al., 2014).

To maximize biological inferences obtained from NGS *ITS2* data sets, we present SymPortal, a new analytical framework for resolving Symbiodiniaceae taxa using NGS data of the *ITS2* marker gene. Our methodology augments tried‐and‐tested intragenomic resolution theory with the power of NGS to resolve between symbiont taxa at a level far surpassing alternative methodologies using this marker. We employ novel logic to identify within‐sample informative intragenomic sequences, which we have termed 'defining intragenomic variants' (DIVs), and use combinations of these DIVs to identify *ITS2* type profiles representative of putative Symbiodiniaceae taxa. This approach means that SymPortal is better able to differentiate between intragenomic and intergenomic sources of *ITS2* variation without the need for more resolute markers, such as the *psbA* ^ncr^. Here we give an overview of the SymPortal analytical framework and demonstrate its ability to achieve superior *ITS2*‐based taxonomic resolutions within the family Symbiodiniaceae. We analyse NGS *ITS2* data in the SymPortal framework and compare the results with the most commonly used approach, the 97% similarity OTU analysis.

## 2 MATERIALS AND METHODS

This section provides an overview of the SymPortal analytical framework before detailing the approach used in the methodological comparison of the SymPortal and 97% similarity OTU analyses. More detailed and continually updated documentation of the SymPortal framework can be found on GitHub (https://github.com/didillysquat/SymPortal_framework).

### 2.1 SymPortal and the reorganization of the former genus *Symbiodinium*

The former genus *Symbiodinium* has recently undergone a revision in systematics to the taxonomic level of family and named Symbiodiniaceae (LaJeunesse et al., 2018). The phylogenetic groupings commonly referred to as ‘Clades’ are now recognized as different genera. Currently seven, about half of the total, are given formal names. The group designated Clade A retains the genus name *Symbiodinium*. Thus, any mention of *Symbiodinium* refers to taxa in Clade A. As pertains to this research, Clade C is now the genus *Cladocopium.*

Of note, the SymPortal framework was written before this taxonomic reorganization and all analyses to date have been completed according to the old organizational system/taxonomy. Given the need for backwards comparability to previous studies, the SymPortal framework will maintain its use of the clade systematics whilst working towards the additional incorporation of the Symbiodiniaceae systematics to ensure forward comparability. The term and taxonomic level of ‘clade’ will therefore still be referenced in the explanation of the SymPortal framework below, and it should be clear that this refers to the phylogenetic groupings of the former genus *Symbiodinium* (Clades A–I).

### 2.2 Overview of the SymPortal analytical framework

The SymPortal analytical framework consists of two parts: the SymPortal analysis and an SQL database with which the analysis is integrated. The analysis may be run either remotely via submission to SymPortal.org or locally. The main difference between these two modes of operation is the database used for the analysis. If running the analysis via submission to SymPortal.org, the remotely hosted SymPortal database is used, while analyses run locally make use of a user‐constructed database. Please see Sections 2.3.4: Accessing the SymPortal analytical framework and 2.3.5: The SymPortal analytical framework's database below for further details.

**Figure 1:**
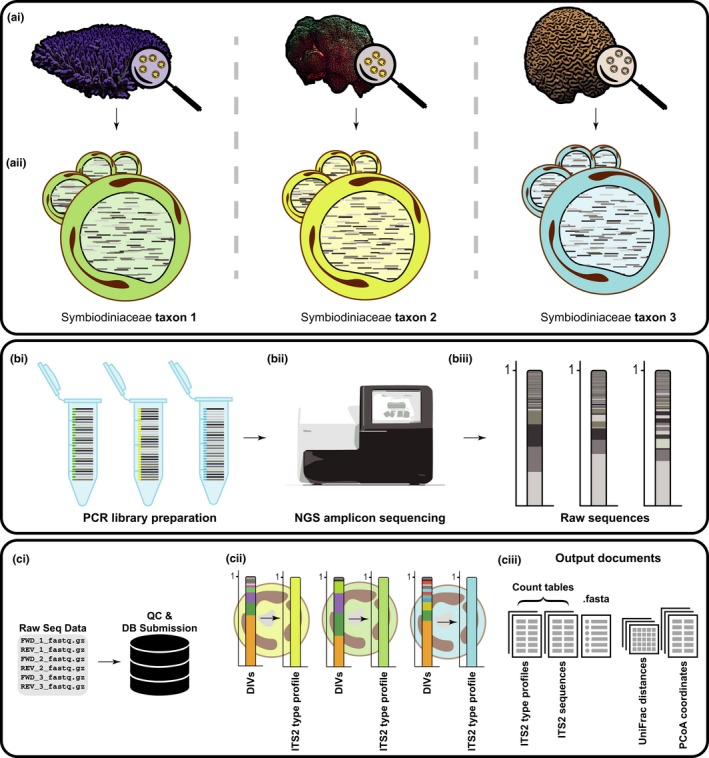
Overview of the typical workflow employing the SymPortal analytical framework. Sampling: (ai) Field sampling: corals containing populations of Symbiodiniaceae are sampled. (aii) Each Symbiodiniaceae cell contains hundreds of copies of the *ITS2* gene representing many unique sequences (intragenomic sequence diversity). In this example, three corals are sampled, each containing a single genetically differentiated population of Symbiodiniaceae*.* PCR library preparation and sequencing: (bi) genomic DNA is extracted from each sample, the *ITS2* region is PCR‐amplified and PCR libraries are prepared for sequencing through clean‐up and adapter ligation. (bii) Prepared libraries are sequenced on the Illumina platform producing, (biii) raw sequencing reads in paired.fastq.gz format. SymPortal analysis: (ci) paired.fastq.gz files are quality control processed and loaded into the SymPortal framework's database before being run through the SymPortal analysis. (cii) During the analysis, re‐occurring sets of *ITS2* sequences are identified as ‘defining intragenomic [*ITS2* sequence] variants’ (DIVs) that are used to define novel or pre‐existing, taxa‐representative, *ITS2* type profiles. (ciii) tab‐delimited count tables are output detailing the abundance of predicted *ITS2* type profiles and post‐quality control sequences found in each sample. A .fasta is also provided detailing nucleotide sequence information. Finally, clade‐separated UniFrac distance matrices and calculated principal coordinate analysis (PCoA) coordinates are output for between‐sample and between‐*ITS2* type profile pairwise comparisons [Colour figure can be viewed at wileyonlinelibrary.com]

Within the SymPortal framework, the SymPortal analysis works by employing the usually substantial *ITS2* intragenomic diversity harboured within every Symbiodiniaceae genome, and captured by NGS *ITS2* sequencing, to resolve between genetically differentiated taxa (Figure 1). The analysis does this by identifying specific sets of ‘defining intragenomic [*ITS2* sequence] variants’ (DIVs) that represent the taxonomic unit of SymPortal, the ‘*ITS2* type profile’ (a term derived from the ‘*ITS2* types’ defined by ‘*ITS2* profiles’ in DGGE‐based methodologies). The database with which the SymPortal analysis is integrated stores sequencing information from every previously conducted SymPortal analysis. With continued use, the SymPortal framework will therefore accrue a catalog of *ITS2* type profiles representative of identified putative Symbiodiniaceae taxa.

Whilst several different Symbiodiniaceae taxa may be harboured by an individual coral, commonly only a single Symbiodiniaceae taxon is found to be predominant (Baums, Devlin‐Durante, & Lajeunesse, 2014; Goulet & Coffroth, 2003; Pettay, Wham, Pinzon, & Lajeunesse, 2011; Thornhill, Xiang, Fitt, & Santos, 2009). In cases where multiple Symbiodiniaceae are harboured by a single coral, these taxa are usually from different genera. Nevertheless, although rare, individual corals may harbour multiple Symbiodiniaceae taxa from the same genera. SymPortal must therefore be able to differentiate between intra‐ and intergenomic sources of *ITS2* sequence variants returned from sample amplicon libraries (Arif et al., 2014; Cunning et al., 2017; Thornhill et al., 2007). To achieve this, SymPortal applies a central principle: the probability that a given set of *ITS2* sequences found in a single coral sample are representative of a single Symbiodiniaceae genotype increases with the number of samples that set is found in (Figure 1c)*.* Key to this principle is that although distinct coral colonies may contain DNA from millions of individual Symbiodiniaceae cells, when considered on a genus by genus basis (*ITS2* sequences from Symbiodiniaceae taxa of different genera are genetically diverse and mostly unalignable), the vast majority of the cells are usually of a single genotype (Baums et al., 2014; Goulet & Coffroth, 2003; Pettay et al., 2011; Thornhill et al., 2009). On the rare occasions when multiple taxa belonging to the same genus are found within a single sample, these taxa may be successfully resolved using SymPortal when each of them is present in other samples as standalone taxa. This principle has been used in DGGE‐based methodologies to successfully identify genetically distinct Symbiodiniaceae taxa that have been verified by additional genetic markers (LaJeunesse & Thornhill, 2011; LaJeunesse et al., 2014; Sampayo et al., 2009).

Given the above approach to differentiating between intra‐ and intergenomic sources of variation, SymPortal's ability to accurately identify DIVs and therefore *ITS2* type profiles representative of Symbiodiniaceae taxa will increase as more samples are incorporated into the analysis. Therefore, to maximize the confidence with which each analysis identifies *ITS2* type profiles indicative of putative taxa, SymPortal makes use of the sequencing information stored in its database from previously run analyses. By having more samples at its disposal, from which it may search for re‐occurring sets of *ITS2* sequences, SymPortal's power to resolve within Symbiodiniaceae increases with use.

### 2.3 The SymPortal workflow

The processes underlying data submission and analysis in the SymPortal analytical framework are documented in detail online at the GitHub wiki (https://github.com/didillysquat/SymPortal_framework/wiki). Below, we provide an overview of these processes alongside an introduction to the general principles underlying the operation of SymPortal.

#### 2.3.1 Sample input

A data submission to SymPortal is typically in the form of a batch of samples in which each sample's sequencing information is represented by a pair of demultiplexed .fastq.gz files (one file containing the forward read and one file containing the reverse read; Figure 1). Amplicon libraries must have been amplified with Symbiodiniaceae *ITS2*‐specific primers. We recommend use of the SYM_VAR primer pair (SYM_VAR_REV/SYM_VAR_5.8S2; Hume et al., 2013; Hume et al., 2015, respectively; Hume, Ziegler et al., 2018), but any amplicon is appropriate as long as the amplicon produced by the SYM_VAR primer pair is nested within it (e.g. ITSintfor2/ITS‐Reverse as used in Arif et al., 2014; Coleman, Suarez, & Goff, 1994; LaJeunesse, 2002; Ziegler et al., 2017). For a comparison of commonly used primer pairs, please refer to Hume, Ziegler et al. (2018).

To maintain standardization between data submissions, all sequence quality control (QC) is done within the SymPortal framework. This is currently conducted using mothur 1.39.5 (Schloss et al., 2009), the blast+ suite of executables (Camacho et al., 2009), minimum entropy decomposition (MED; Eren et al., 2015) and custom functions written in Python to minimize the incorporation of artefactual and non‐Symbiodiniaceae sequences, while increasing the informative potential of each library.

Post‐QC, the sequencing information from each sample is loaded into the SymPortal database, at which point it is available for subsequent analyses (see Section 2.3.5: The SymPortal analytical framework's database below).

#### 2.3.2 An analysis: Searching for *ITS2* type profiles and identifying DIVs

**Figure 2:**
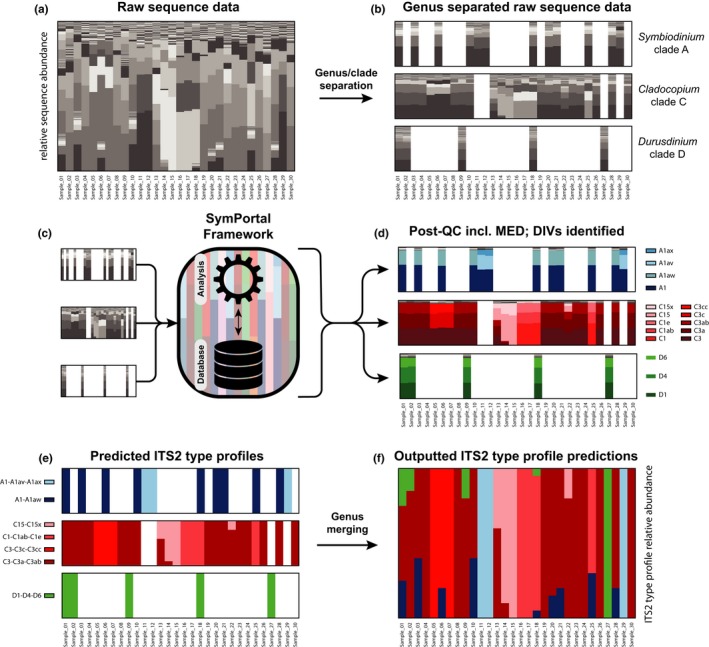
Determination of *ITS2* type profiles in samples containing mixed Symbiodiniaceae communities. In all subplots, each stacked bar represents sequence data or predicted *ITS2* type profiles for a single given sample with individual bars of the stack representing relative abundances within the sample. Coloured bars represent specific *ITS2* sequences or *ITS2* type profiles. Grey scale bars represent unidentified *ITS2* sequences. (a) Samples that contain *ITS2* sequence diversity originating from intergenomic and intragenomic variation are submitted to SymPortal. (b) Raw sequences are separated by genus as part of the standard quality control pipeline. (c–e) The remaining analysis is conducted on the genus‐separated sequence data, but only genus‐separated collections of more than 200 sequences (per sample) are searched for *ITS2* type profiles to minimize sequencing depth artefacts. Multiple *ITS2* type profiles of the same genus (e.g. C15‐C15x and C3‐C3a‐C3ab) may be predicted within individual samples, if each of the *ITS2* type profiles are found in isolation in the current or previously analysed data sets. (f) Before output, the genus‐separated predicted *ITS2* type profiles are merged and abundances are adjusted relative to preseparation proportions. The data used in this figure have been generated for the purpose of illustration and are not based on actual data. The code used to generate the data can be found at https://github.com/didillysquat/sp_ms_figure_creation [Colour figure can be viewed at wileyonlinelibrary.com]

Each new SymPortal analysis makes use of the sequencing information associated with not only the new samples, but all previously analysed samples as well. During an analysis, each sample's collection of *ITS2* sequences is algorithmically searched in a genus‐/clade‐separated manner (Figure 2) to identify the largest set of *ITS2* sequences that also occur in other samples in the analysis. To minimize the effects of sequencing depth artefacts, these genera‐separated collections of sequences will only be searched if they contain more than 200 sequences. When a set of *ITS2* sequences is found to re‐occur in a sufficient number of samples, each of these sequences is considered to be a DIV and these DIVs are used to characterize an *ITS2* type profile (Figures 1 and 2). If for any given sample a re‐occurring set of DIVs cannot be found, then an *ITS2* type profile will be assigned to this sample that is defined simply by the sample's most abundant *ITS2* sequence.

When identifying *ITS2* type profiles, the presence as well as the abundance of characterizing DIVs are considered. For each set of supported *ITS2* sequences that is found in the algorithmic search, each constituent *ITS2* sequence is tested to see whether it has a multimodal distribution across the samples in which the *ITS2* set of sequences was found (i.e. whether there are two or more distinctly different abundances of the sequence). If a multimodal distribution is identified, the collection of samples to which the *ITS2* set of sequences were associated is split according to the distribution of the sequences between the different modes. Once no further multimodal distributions are identified, the *ITS2* sequences of the separated sets of *ITS2* sequences are considered DIVs. In this way, it is possible for different ITS2 type profiles to contain exactly the same collection of DIVs but at different abundances. At this point, the relative abundances of the *ITS2* type profile's DIVs within all the samples they were found in are then used to characterize the *ITS2* type profile. Therefore, when searching for *ITS2* type profiles within samples, a match is only considered if all of the characterizing DIVs are found, and these have relative abundances that fall within the abundance ranges determined during characterization of the given *ITS2* type profile.

#### 2.3.3 Mixed Symbiodiniaceae community samples

Individual samples may contain taxa from more than one genus in the family Symbiodiniaceae (formerly Clades A–G; LaJeunesse et al., 2018). Between these genera, *ITS2* sequences are mostly unalignable. Therefore, as a precursor to all analyses, sequence data are separated by genus/clade before subsequent analysis of *ITS2* type profiles. The genus‐/clade‐separated data are then merged back together before final output with abundances of predicted *ITS2* type profiles adjusted relative to the preseparation proportions (Figure 2).

Samples may also contain multiple Symbiodiniaceae populations from the same genus, and each can make up a considerable proportion of the host's algal symbiont complement. SymPortal is able to identify multiple *ITS2* type profiles of the same genus within a given sample as long as each of the *ITS2* type profiles has been found as the sole *ITS2* type profile in another sample, such as C3‐C3a‐C3ab and C15‐C15x in Figure 2 (see Section 2.3.6, SymPortal output, for an explanation of *ITS2* type profile systematics).

#### 2.3.4 Accessing the SymPortal analytical framework

The SymPortal analytical framework may be run either remotely, through submission of data to a version of the framework hosted remotely at SymPortal.org, or locally, by running the Python scripts housed on the GitHub repository (https://github.com/didillysquat/SymPortal_framework). Instructions for submitting data to the remote instance of the framework are detailed at the GitHub wiki and at SymPortal.org. Additionally, policies regarding data ownership and accessibility when submitting to the remote instance of SymPortal are also hosted on SymPortal's GitHub wiki. Analyses run remotely through SymPortal.org will have access to the latest version of the SymPortal database. This remotely hosted PostgreSQL database contains sequencing information from all samples previously analysed that were submitted via SymPortal.org. Analyses run via SymPortal.org will therefore have access to the greatest possible resolving power and enable comparability to other data sets previously submitted to SymPortal.org. If running the SymPortal framework locally, it will be necessary for the user to populate the local database with which the local SymPortal analysis will integrate. As such, when running analyses locally, the ability to resolve *ITS2* type profiles will be contingent on the extent of sequencing information housed in the local database. Further documentation may be found at the SymPortal GitHub repository.

#### 2.3.5 The SymPortal analytical framework's database

Irrespective of whether analyses are run locally or remotely via SymPortal.org, the sequencing information of every data set analysed by SymPortal is stored in the associated database. This sequencing information is stored when a data set is loaded into a SymPortal database instance (Table 1) and when analyses are run (Table 2). Remotely run analyses will use the remote SymPortal database, while local analyses will use a locally defined database. All database instances are integrated into the Python‐scripted analysis through the Django API (https://www.djangoproject.com/). In this way, the sequencing information from all previously analysed samples is accessible for use in future analyses.

**Table 1:** Database tables populated upon loading of a new data set into the SymPortal framework

Table name	Table description
DataSet	Equivalent to a set of samples loaded into, and analysed on, the SymPortal framework. Contains fields to store basic information such as: the submitting user, the working directory, or the data set's unique name
DataSetSample	Equivalent to a single sample from a specific data set. Has a foreign key relation to the DataSet table. Contains fields that store quality control statistics, as well as basic sample information, for example name of samples, number of reads after making contiguous sequences, or number of nodes after minimum entropy decomposition
CladeCollection	An abstract object that is a collection of all DataSetSampleSequence instances for a specific DataSetSample that are all of the same clade and have a total abundance > 200. Has a foreign key relationship to DataSetSample. Contains fields that store basic information on the DataSetSampleSequence instances in the collection and of the clade^a^
DataSetSampleSequence	Equivalent to a single unique post‐QC sequencing read returned from a given data set sample. Contains a field for abundance. Has foreign key relationships to ReferenceSequence, CladeCollection and DataSetSample
ReferenceSequence	Equivalent to a single unique post‐QC sequencing read, independent of any one sample. Contains fields for name, sequence, clade and accession number

a The clade system of taxonomic naming within the former genus *Symbiodinium* has now been superseded by new family‐ and genera‐level descriptions (LaJeunesse et al., 2018). In the interest of maintaining reverse comparability to previous studies, SymPortal will continue to relate its outputs to the clade divisions. In time, SymPortal will also be updated to include the new Symbiodiniaceae organization.

**Table 2:** Database tables populated during a SymPortal analysis

Table name	Table description
DataAnalysis	Equivalent to a single SymPortal analysis. Contains fields to store high‐level information about the analysis, such as DataSet objects included in the analysis, name, description and parameters used within the analysis
AnalysisType	Equivalent to an *ITS2* type profile. Contains fields relating to *ITS2* type profile features, for example the ReferenceSequence objects that characterize the AnalysisType (DIVs) listed in order of abundance, the ReferenceSequence objects that are found as the most abundant sequences in each of the samples in which the AnalysisType is found, a list of the CladeCollection objects in which the AnalysisType is found, or the maximum and minimum relative abundances of the DIVs that characterize the AnalysisType. Has a foreign key relationship to DataAnalysis
CladeCollectionType	An abstract table used to link the database tables associated with data analyses to the database tables associated with data set loading. Has foreign key relationships to CladeCollection and AnalysisType^a^

a The clade system of taxonomic division within the former genus *Symbiodinium* has now been superseded by new family‐ and genera‐level descriptions (LaJeunesse et al., 2018). In the interest of maintaining reverse comparability to previous studies, SymPortal will continue to relate its outputs to the clade divisions. In time SymPortal will also be updated to include the new Symbiodiniaceae organization.

In addition to storing detailed sequencing information for each submitted data set, the database also stores details of all completed analyses (Table 2). Analysis details, such as which *ITS2* type profiles were found and which DIVs characterize those profiles, are associated with the respective sequencing information, e.g. which samples the ITS2 type profiles were found in, and which of the samples' sequences were identified as DIVs. The remotely hosted SymPortal database therefore represents a powerful analytical and reference resource, effectively accruing a catalogue of global Symbiodiniaceae diversity as the number of samples analysed grows.

With every new data set submitted to the SymPortal framework's database, the amount of sequencing information available for use in the SymPortal analysis will increase. Effectively, each sequential analysis will have access to a more resource‐rich version of the database. In this way, subtle differences between the identities of the *ITS2* type profiles discovered in consecutive analyses may occur as SymPortal's power to resolve improves. As such, when comparing outputs from two different SymPortal analyses, it will be helpful to consider which data sets from the SymPortal database were included in the given analyses. For this reason, every analysis count table output will provide the unique database identifier (UID) of the analysis it is related to. From this information, a list of the data sets that were incorporated into the analysis may be generated. However, changes that occur between analyses run against sequential database versions are likely to be small. Samples from different analyses that contain similar *ITS2* sequence diversities will still have comparable *ITS2* type profile designations. At the time of writing, the latest analysis (DataAnalysis UID 44) contained 7,173 samples, representing the sampling efforts of 35 studies (35 DataSet objects). Within these samples, 771 different *ITS2* type profiles were identified, representing 10,539 unique *ITS2* type profile/sample associations. Importantly, 50% of these unique associations are represented by the 92 most abundant *ITS2* type profiles (Figure S1). A summary of the database tables held in the SymPortal framework's database is given in Tables 1 and 2.

#### 2.3.6 SymPortal output

The SymPortal output consists of five core output files. Two tab‐delimited count tables each report on post‐QC sequences, *ITS2* type profile abundances identified in the output's samples (each reported in absolute and relative abundances) and a .fasta file containing nucleotide sequence information for every *ITS2* sequence reported in the count table. In addition, a set of genus‐separated Bray–Curtis‐ or UniFrac‐based distance matrices for between‐sample and between‐*ITS2* type profile comparisons, and principal coordinate analysis (PCoA) coordinates for each of these distance matrices, are output. To give an overview of data submissions and data analyses, plots of sequences and *ITS2* type profile abundances across samples as well as graphical representations of the PCoA results are additionally generated for the user (see Figures S2 and S3 as an example).

For both count tables (i.e. sequences and *IT2* type profiles), the format resembles the OTU count tables commonly used in 16S analyses. Therefore, the format of these tables is ideally suited to be used as direct input to commonly implemented statistical analyses for sequencing diversity and abundance data. The SymPortal outputs for the data analysed in this study is provided as Files S1–S11.

The sequence output table lists the sequences found in order of their abundances across all the samples included in the output (Files S1 and S2). For each sequence, its abundance in each of the samples is reported. A GenBank accession number (if available) or alternatively a UID is also provided for each *ITS2* sequence that allows it to be identified in the SymPortal database. In this way, a single definitive source for the *ITS2* sequence is always available. The nucleotide sequence for each of the *ITS2* sequences reported in the count table is available in a .fasta file (File S3). Finally, for each sample, the number of sequences retained at each step of QC is reported.

The *ITS2* type profile abundances are output in decreasing order of the number of samples they were found in for the current analysis output (Files S4 and S5). For each *ITS2* type profile, several features are reported as summarized in Table 3.

**Table 3:** *ITS2* type profile count table output features

Count table feature (table header name)	Description
*ITS2* type profile UID	A unique database identifier number that uniquely identifies each *ITS2* type profile in the SymPortal database
Clade	The clade (A–I) of the *ITS2* type profile^a^
Majority *ITS2* sequence	The sequence(s) that was(were) identified as the most abundant in each of the samples that the type profile was found in
Associated species	A list of any Symbiodiniaceae species descriptions that the given *ITS2* type profile could relate to
*ITS2* type profile abundance local	The number of samples in the output that the given *ITS2* type profile was found in
*ITS2* type profile abundance DB	The number of samples in the entire database that contained the *ITS2* type profile in question
*ITS2* type profile	The name of the *ITS2* type profile in question
Sequence accession/SymPortal UID	Either an accession number or SymPortal UID for each of the DIV sequences that define the *ITS2* type profile in question
Counts	Number of sequence reads for DIVs of a given *ITS2* type profile in a given sample

a The clade system of taxonomic naming within the former genus *Symbiodinium* has now been superseded by new family‐ and genera‐level descriptions (LaJeunesse et al., 2018). In the interest of maintaining reverse comparability to previous studies, SymPortal will continue to relate its outputs to the former clade divisions. In time, SymPortal will also be updated to include the new Symbiodiniaceae organization.

To aid in relating the determined *ITS2* type profiles to species descriptions, a list of associated Symbiodiniaceae species is reported for each *ITS2* type profile where applicable (Table 3). The purpose of this list is to aid the researcher in identifying potential Symbiodiniaceae species that the *ITS2* type profile could represent. For a species to be associated with an *ITS2* type profile, the *ITS2* sequence(s) characteristic of the species, as detailed in its description, must all be found in the list of DIVs that define the *ITS2* type profile in question. New species information will be added to the SymPortal database, and therefore associated with outputs, as new descriptions are made. Importantly, this is not a list of species that the *ITS2* type profile definitely represents, and it remains the responsibility of the researcher to carefully consider the species descriptions to assess whether the queried sample may be the species in question. Depending on the description, this may require the use of further genetic, morphological, or physiological evidence.

The naming scheme of the *ITS2* type profiles is informative. It denotes which underlying DIVs the *ITS2* type profiles are composed of in decreasing order of abundance. Hence, the *ITS2* type profile ‘C3‐C3gulf‐C3c‐C3aq’ contains DIVs that are *ITS2* sequences denoted as C3, C3gulf, C3c and C3aq. This nomenclature also provides information on which of the DIVs were the most abundant in each of the samples in which the *ITS2* type profile was found. In the case of ‘C3‐C3gulf‐C3c‐C3aq,’ the C3 sequence was the most abundant sequence in all of the samples in which this *ITS2* type profile was found, as this DIV is listed first in the name. Interestingly, a Symbiodiniaceae taxon represented by an *ITS2* type profile is sometimes characterized by more than one most abundant sequence. For example, in the type ‘C3/C3c‐C3gulf’ some of the samples in which this *ITS2* type profile was found contained the C3 sequence as the most abundant DIV, but in others the C3c sequence was most abundant. This comajority abundance is denoted by the ‘/’ in the *ITS2* type profile name.

Distance matrices of pairwise comparisons are output from SymPortal to aid in the quantification of similarity or relatedness between study samples (Files S8 and S10) and *ITS2* type profiles (File S6), respectively. Given the inability to align some *ITS2* sequences from different genera within the Symbiodiniaceae, pairwise differences are calculated for genus‐separated groupings of *ITS2* sequences. Distance matrices are calculated based on either a Bray–Curtis method (sensu Smith, Vaughan, Ketchum, McParland, & Burt, 2017c) or using the weighted UniFrac method (Lozupone, Lladser, Knights, Stombaugh, & Knight, 2011). UniFrac is often used to compare bacterial communities by assessing similarity between sets of 16S rDNA sequences representative of bacterial taxa (Lozupone et al., 2011). As such, it is well suited to comparing similarities between sets of *ITS2* sequences where closely related *ITS2* type profiles (those sharing a most abundant *ITS2* sequence) are likely to be more closely related, the more similar their *ITS2* sequence complements are. To compare similarity between two sets of sequences, UniFrac calculates the proportion of sequences from each set that share a branch on a precomputed phylogenetic tree containing all sequences from both sequence sets. Such a phylogenetic tree is therefore required for the analysis and computed as detailed in the following.

Weighted UniFrac distance matrices are calculated in mothur 1.39.5 (Schloss et al., 2009). A bootstrapped neighbour‐joining (NJ) tree is used as input to the unifrac.weighted command and generated using a combination of: mafft (Katoh & Standley, 2013) to create a multiple sequence alignment, seqboot from phylip (http://evolution.genetics.washington.edu/phylip/) to generate multiple alignments by resampling, mothur's implementation of clearcut (Sheneman, Evans, & Foster, 2006) to generate multiple trees, and sumtrees (http://dendropy.org/programs/sumtrees.html) to generate a 50% majority rule consensus tree.

### 2.4 Comparison of SymPortal's resolution to that of a 97% similarity cutoff clustering approach

**Figure 3:**
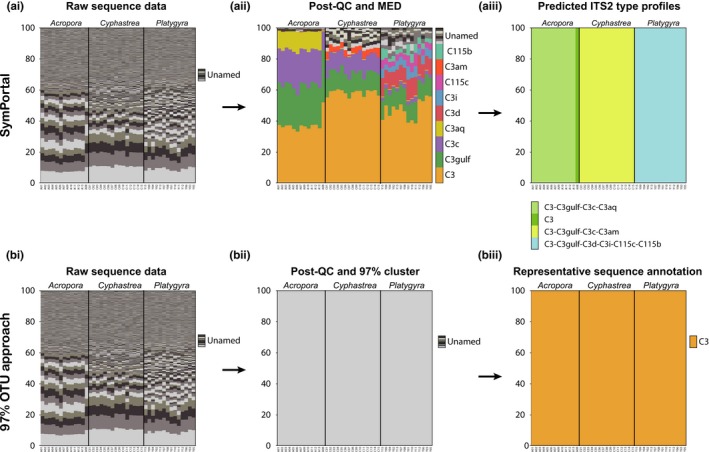
Comparison of the SymPortal analytical framework (top) with operational taxonomic unit (OTU) generation based on 97% similarity cutoff clustering (bottom) from the Smith, Ketchum et al. (2017b) data set (three coral species with 13–15 samples for each coral species). The comparison demonstrates the ability of both methods to resolve between closely related (i.e. the same most common *ITS2* sequence) Symbiodiniaceae taxa. From left to right, plots represent progress in each analysis: (ai, bi) raw sequence data, (aii) post‐quality control (QC) and minimum entropy decomposition analyses (MED), (bii) post‐QC and 97% similarity clustering, (aiii) determination of *ITS2* type profiles through the identification of defining intragenomic sequence variants (DIVs), and (biii) identification and annotation of representative OTUs. In all plots, each stacked bar column represents a single sample with each individual bar representing either the proportion of a single *ITS2* sequence relative to the total abundance of sequences in that sample or the relative abundance of *ITS2* type profiles identified in each sample (plot aiii only). Notably, SymPortal *ITS2* type profiles determine distinct Symbiodiniaceae taxa associated with distinct hosts, whereas a 97% OTU approach suggests the same symbiont is associated with all hosts. Coloured bars denote DIV sequences (see coloured key) as opposed to grey scale bars that represent non‐DIV sequences [Colour figure can be viewed at wileyonlinelibrary.com]

To compare the ability of SymPortal to resolve putative Symbiodiniaceae taxa with that of the most commonly used *ITS2* NGS analysis approach, the 97% similarity OTU approach, we reanalysed data from a previous study with both methods (Figure 3).

#### 2.4.1 The test data set

The test data set was collected as part of Smith, Ketchum et al. (2017b). It consisted of Symbiodiniaceae *ITS2* and *psbA* ^ncr^ NGS amplicon data from 13, 15 and 14 coral samples from each of three coral species: *Acropora downingi*, *Cyphastrea microphthalma* and *Platygyra daedalea*, respectively. All samples, from each of the coral species, were found to contain Symbiodiniaceae taxa of the species *Cladocopium thermophilum* (Hume et al., 2015; LaJeunesse et al., 2018) by Smith, Ketchum et al. (2017b). Briefly, corals were collected from Saadiyat reef (24°35′56.4″N, 54°25′17.4″E) in the southern Persian/Arabian Gulf in February 2016. DNA was extracted through: sample lysis in an sodium dodecylsulphate‐based lysis buffer, binding of DNA on carboxylated modified SeraMag beads (GE Healthcare Life Sciences), washing in 80% ethanol and subsequent dissolution in molecular‐grade water. Amplicon libraries were generated using Symbiodiniaceae‐specific primer pairs for *ITS2*, SYM_VAR_5.8S2 and SYM_VAR_REV (Hume et al., 2013, 2015; Hume, Ziegler et al., 2018) and for *psbA* ^ncr^, PSBA_NC_F and PSBA_NC_R (Smith, Ketchum et al., 2017b). Amplicon libraries were prepared for, and sequenced on, the Illumina MiSeq platform. For the sake of clarity in demonstrating each approach's analysis, only sequences of the genus *Cladocopium* (formerly Clade C; LaJeunesse et al., 2018) were considered in the comparison (although samples contained some sequences from the genus *Symbiodinium*, formerly Clade A). Of note, the figures automatically generated during the SymPortal data analysis conducted for this study that contain genus *Cladocopium* and *Symbiodinium* information are provided in Figures S2 and S3.

The availability of both *ITS2* and *psbA* ^ncr^ NGS amplicon data in this data set makes it ideal for testing *ITS2*‐based genetic resolutions. *ITS2*‐resolved genetic delineations can be verified against phylogenies made from the highly variable, but less intragenomically diverse *psbA* ^ncr^ marker. Smith, Ketchum et al. (2017b) have used the metahaplotype approach to resolve the samples in this data set into three groups that corresponded to the respective coral host origin. Critically, however, the metahaplotype approach has no means of differentiating between within‐sample intra‐ and intergenomic sources of *ITS2* diversity. Rather, it is reliant on the *psbA* ^ncr^ marker to verify that only a single dominant taxon is present in each sample prior to application of the *ITS2*‐based ordination resolution approach (Smith, Ketchum et al., 2017b).

#### 2.4.2 Sample analysis using generation of OTUs by 97% similarity cutoff clustering

Clustering to OTUs at the 97% similarity cutoff threshold is an analytical approach originating from the analysis of 16S rDNA in bacterial ecological studies (Stackebrandt & Goebel, 1994; Vetrovsky & Baldrian, 2013). This approach was applied to Symbiodiniaceae *ITS2* DNA to collapse intragenomic sequence variability and to be able to resolve ecologically discrete entities (Arif et al., 2014). The 97% threshold was applied to Symbiodiniaceae due to its documented ability to collapse all *ITS2* diversity in monoclonal cultures of Symbiodiniaceae (Arif et al., 2014). In the approach described by Arif et al. (2014), sequences recovered from all samples are split by genus, pooled and clustered into OTUs. Representative sequences are then associated with each of the OTUs. More recently, a modification to this method was proposed by Cunning et al. (2017) in which sequences are still clustered at a 97% similarity cutoff but on a sample‐by‐sample basis rather than across all samples. This more recent approach was developed to address the fact that the most abundant *ITS2* sequences from different Symbiodiniaceae species may be more similar to one another than the intragenomic variants found within those species (Arif et al., 2014; Cunning et al., 2017). The representative OTUs returned from this approach will usually be equivalent to the most abundant sequence in each sample per genus. To maintain the maximum potential for resolution between the samples in our test data set, we undertook both analyses, namely within and across sample clustering approaches.

For both clustering methods, the initial QC of sample sequences was the same as the mothur‐based component of QC used in SymPortal. Briefly, mothur_1.39.5 (Schloss et al., 2009) was used to create contigs from paired forward and reverse demultiplexed .fastq.gz files using the *make.contigs* command. On a sample‐by‐sample basis, the following sequence of commands was then applied. The *screen.seqs* command was used (*maxambig = 0, maxhomop = 5*) to discard sequences putatively generated from sequencing errors. The resultant .fasta file was used as an input for *blastn* with the *max_target_seqs* argument set to 1 and an output format string of ‘*6 qseqid sseqid evalue pident gcovs*’ (Camacho et al., 2009). A custom blast database was used that contained a single representative sequence for each of the nine clades of the former genus *Symbiodinium* (Clades A–I; LaJeunesse et al., 2018; File S12). Any sequences not returning the Clade C entry as the closest match were disregarded from the analyses (see above). The *unique.seqs* command was used to create a nonredundant collection of sequences represented by a .name and a .fasta file. The remainder of the QC was performed using both the .name and the .fasta files produced. Next, *split.abund* was run (*cutoff = 2*) to discard sequences that were not found at an abundance > 2 in each sequenced sample – again, to reduce incorporating sequences with sequencing errors. The *pcr.seqs* command (*pdiffs = 2, rdiffs = 2*) was used to trim the primer sequence regions from the returned sequences. In addition, this command was used to discard sequences in which the specific primer pairs could not be found – indicative of poor sequencing quality – allowing a deviation of ≤2‐nucleotide differences in either of the forward or the reverse primer sequences. Sequences were again made nonredundant using the *unique.seqs* command, then sequences <184 bp and >310 bp in length were removed using the *screen.seqs minlength* and *maxlength* parameters. Sequences were again made nonredundant.

To perform clustering on pooled sequences across all samples, for each of the .fasta and .name file pairs created above (one pair per sample), a single redundant .fasta file was created using the *deunique.seqs* command. Each of these deuniqued .fasta files was then combined to create a single redundant .fasta file containing all post‐QC sequences from the sequencing effort. A single master nonredundant .fasta and .name file set was then created using the *unique.seqs* command. This .fasta file was then aligned using mafft (Katoh & Standley, 2013) with the ‐‐auto flag active. A phylip style distance matrix was created using mothur's *dist.seqs* command with *countends = F* and *output = lt.* The sequences were then clustered at a 3% dissimilarity cutoff using the average‐neighbour algorithm, the phylip distance matrix and the .name file using the *cluster* command (*method = average, cutoff = 0.03*) to produce OTUs. Finally, the *get.oturep* command was used to return the most abundant sequence as a representative sequence for each OTU generated by the clustering.

To perform clustering on sequences within samples: The same process as above was used to align, cluster and generate representative OTU sequences, only working on a sample‐by‐sample basis with the respective pairs of nonredundant .fasta and .name files.

#### 2.4.3 Sample analysis using SymPortal's framework

Paired .fastq.gz files for each sample were run through a SymPortal analysis in one batch. The abundances of the *ITS2* type profiles belonging to Clade C, as reported in the SymPortal output count table, were directly used to create Figure 3. Output count tables are provided as Files [Link], [Link], [Link], [Link], [Link].

#### 2.4.4 Plotting of the *psbA* ^ncr^ maximum‐likelihood (ML) phylogeny

To create a *psbA* ^ncr^ phylogeny, the preprocessed *psbA* ^ncr^ sequences from the data associated with Smith, Ketchum et al. (2017b) were used to generate a single consensus sequence for each sample. Generation of these consensus sequences is detailed at https://github.com/didillysquat/sp_ms_figure_creation/blob/master/SP_MS_figure_making.ipynb. Unfortunately, *psbA* ^ncr^ marker amplicons were not available for one of the *Platygyra* samples, Y11, due to an indexing error (Y11 was not included in the original study; Smith, Vaughan et al., 2017c). A multiple sequence alignment was created from these sequences (File S13). A best nucleotide substitution model was selected in mega7 (Tamura, Stecher, Peterson, Filipski, & Kumar, 2013) and used to plot a bootstrapped (100 replications) ML phylogeny.

### 2.5 Figure creation

**Figure 4:**
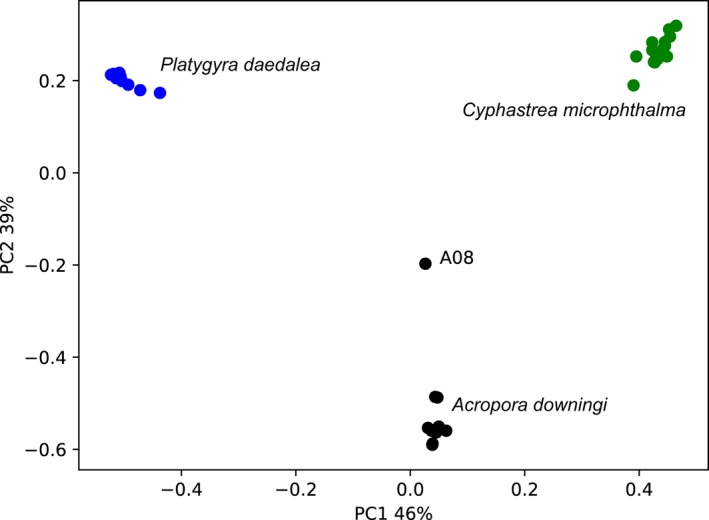
Principal coordinate analysis (PCoA) plot based on UniFrac distances between *Cladocopium* (formerly Clade C; LaJeunesse et al., 2018) *ITS2* sequences found in samples from this study. The plot is made directly from the principal component coordinates output as part of the SymPortal analysis (File S11). Samples are coloured according to host species, as annotated. Sample A08 is separately annotated given its dissimilarity from the main *Acropora* spp. cluster [Colour figure can be viewed at wileyonlinelibrary.com]

**Figure 5:**
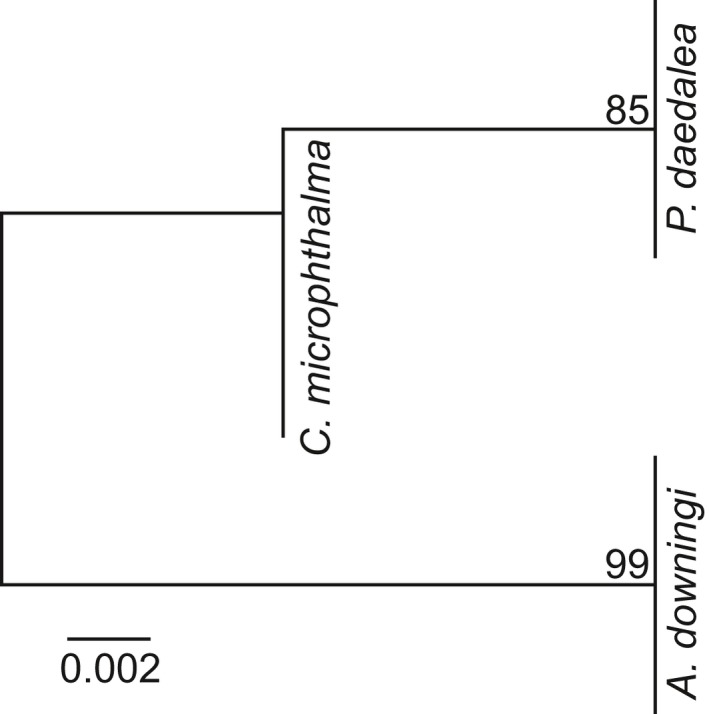
Unrooted maximum likelihood phylogeny based on the *psbA* ^ncr^ sequences from all the samples associated with the Smith, Ketchum et al. (2017b) study. Samples resolved in three positions on the tree according to host species, as annotated. Bootstrap support values < 100 (100 replicates) are annotated

The largely programmatic process used to make figures 2–5 is documented here: https://github.com/didillysquat/sp_ms_figure_creation.

## 3 RESULTS

### 3.1 *ITS2* type profile determination with SymPortal

Running the 42 samples from three coral species (*Acropora downingi*, *Cyphastrea microphthalma* and *Platygyra daedalea*) from the Persian/Arabian Gulf (see Methods) generated four *Cladocopium* *ITS2* type profiles characterized by between one and six DIVs (output count tables for sequences and *ITS2* type profiles are provided in Files S1–S5). Notably, 41 of the 42 samples were represented by three of the four *ITS2* type profiles, which correlated to host genus (Figure 3): *A. downingi*, C3‐C3gulf‐C3c‐C3aq; *C. microphthalma*, C3‐C3gulf‐C3c‐C3am; and *P. daedalea*, C3‐C3gulf‐C3d‐C3i‐C115c‐C115b. The fourth *ITS2* type profile, denoted as ‘C3’ in the SymPortal analysis (Files S4 and S5), was found in only one sample that was collected from an *A. downingi* coral, sample A08. This *ITS2* type profile was most closely related to the *ITS2* type profile found in the other samples from *A. downingi* corals (denoted as C3‐C3gulf‐C3c‐C3aq in the SymPortal analysis, Files S4 and S5). Despite the C3, C3gulf, C3c and C3aq sequences being present, the A08 sample was not assigned to C3‐C3gulf‐C3c‐C3aq due to the C3aq sequence being at a relative abundance that was lower than the defining abundances for the *ITS2* type profile. As no other *ITS2* type profile could be assigned to the sample, it was conservatively assigned the C3 *ITS2* type profile based only on the sample's most abundant *ITS2* sequence from the clade in question (Clade C). Plotting the within‐genus UniFrac distance‐based PCoA coordinates for pairwise sample comparisons (provided as part of the SymPortal output; File S11) also illustrates a clustering of samples according to host species (Figure 4). One *Acropora* sp. sample, A08, falls outside of the main *Acropora* sp. grouping. The cause of this divergence is the difference in *ITS2* diversity (abundance and presence/absence of *ITS2* sequences) contained in this sample compared to the other samples of this host species, as can be seen in Figure 3(aii).

The host‐correlated resolution of the SymPortal analysis is in accordance with the results from the ordination‐based metahaplotype approach of Smith, Ketchum et al. (2017b) that also split the samples into three groups.

### 3.2 *ITS2* OTU generation by 97% similarity cutoff clustering, within and across samples

The within‐ and across‐sample clustering approaches provided identical results. All sequences clustered into a single OTU, which was represented by the C3 sequence (Figure 3; nucleotides 5–267 of sequence KX815267). Clustering into this single OTU was concluded at >98% dissimilarity (i.e. before the 97% threshold could be reached), illustrating the much lower sequence and taxon diversity in comparison to 16S rDNA amplicon studies (Arif et al., 2014; Ziegler et al., 2017).

### 3.3 Agreement between the *ITS2* and *psbA* ^ncr^ genetic markers

Construction of an unrooted ML phylogeny from the *psbA* ^ncr^ sequences associated with the Smith, Ketchum et al. (2017b) data set demonstrated a host‐correlated resolution of the study's samples (Figure 5). This resolution is in accordance with that of the SymPortal results but undetected by the OTU approach. Although not detected in the ML phylogeny, the *psbA* ^ncr^ sequence of sample A08 differed from all other *Acropora*‐derived sequences in four positions (three separate indels). All other *Acropora*‐derived sequences were identical. This difference is also realized in the *ITS2* sequence and type profiles recovered and designated for A08 by the SymPortal analytical framework. Of the 13 *Platygyra* sequences for which *psbA* ^ncr^ sequences were available, four showed a divergence from the consensus (three at a single position and one at two positions; samples Y03, Y04, Y05 and Y06; File S13). This divergence from the consensus was not correlated with a separate *ITS2* type profile designation and when the *ITS2* data for the *Platygyra* samples is ordinated (PCoA from UniFrac‐based distances), separation of these samples from the other *Platygyra* samples is questionable (Figure S4). Only one *Cyphastrea* sample differed from the *psbA* ^ncr^ consensus sequence (one single nucleotide indel).

## 4 DISCUSSION

The multicopy *ITS2* sequence diversity harboured within every Symbiodiniaceae genome represents a wealth of information that is currently underutilized in many NGS analytical approaches. Here, we demonstrate that by exploiting this diversity, the SymPortal analytical framework is able to resolve between closely related Symbiodiniaceae genotypes at a resolution never before achievable with the *ITS2* marker alone (Figure 3). Our results demonstrate that while all sequence diversity was effectively collapsed using a 97% similarity clustering method (as commonly used in current Symbiodiniaceae *ITS2* approaches using NGS data), the SymPortal framework was able to identify four putative and distinct symbiont taxa that were harboured in multiple samples across each of the three coral host species investigated (Figure 3).

While intragenomic *ITS2* sequence analysis has been used in characterizing Symbiodiniaceae taxa in more traditional approaches such as DGGE for some time (LaJeunesse, 2002), the uptake of NGS technologies has afforded researchers the power to interrogate PCR amplicon libraries never previously possible (Arif et al., 2014). In particular, the improvement in sequencing depth and accuracy afforded by NGS makes it the perfect platform for characterizing multicopy marker amplicon diversity. Indeed, we see from the example data set analysed here that the introduction of the concept of using ‘defining intragenomic variants’ (so‐called DIVs in our SymPortal approach) for NGS within the SymPortal analysis is (a) effective in resolving between intra‐ and intergenomic sources of *ITS2* sequence variants (more so than any *a priori* clustering cutoff method or most‐abundant sequence method) and (b) does so at a resolution surpassing classical DGGE approaches. For example, DIVs used to differentiate between different *ITS2* type profiles may be found at relatively low abundances (~<5%; Figure 3). Such sequences would previously have been below detection limits. As such, taxonomic inferences afforded by these DIVs would not have been achievable (Hume et al., 2015; Smith, Hume et al., 2017a).

The informative nature of the intragenomic diversity found within Symbiodiniaceae samples may be put into context by comparing the *ITS2* type profile outputs from the SymPortal analysis, the ordination‐based *ITS2* analytical approaches (the metahaplotype approach of Smith et al. and the UniFrac‐based PCoA groupings of the SymPortal output), and the *psbA* ^ncr^ alignment and phylogeny (Figures 4 and 5; File S13 and Figure S4). The only sample to return an *ITS2* type profile designation different from other samples of the same host origin was sample A08. The visually different *ITS2* sequence profile of this sample (in the ordination and sequence profiles), as well as the conservative C3 *ITS2* type profile designation of SymPortal, are in agreement with its disparate *psbA* ^ncr^ sequence (differing from the consensus by three indels across four nucleotides). Within the other two host species, the maximum divergence from the *psbA* ^ncr^ consensus sequence was by two separate single nucleotide indels. Whilst only one of the *Cyphastrea* samples differed from the consensus, four of the *Platygyra* samples returned different *psbA* ^ncr^ sequences, three of which had the same indel. Although none of these samples were designated as unique *ITS2* type profiles, ordination of the *ITS2* profiles did suggest some grouping of the samples that returned the same *psbA* ^ncr^ indel (Figure S4). A greater number of samples would be required to elucidate the robustness of this resolution, but it would appear that divergence in the *psbA* ^ncr^ (even as small as a single indel) may also be reflected in the *ITS2* marker. Given the hypervariable character of the *psbA* ^ncr^ region in general, and the fact that even closely related taxa may have considerable differences between their most abundant *psbA* ^ncr^ sequence (as evidenced by the between‐host species differences seen here), it would appear that exploiting intragenomic *ITS2* diversity in phylogenetic resolutions within Symbiodiniaceae enables a resolution comparable to that of *psbA* ^ncr^ (at least when considering the short read sequencing‐derived *psbA* ^ncr^ amplicons of the Smith et al. data)*.* Furthermore, because any two *ITS2* sequences from the same Symbiodiniaceae clade (A–I) may be aligned, the taxonomic breadth over which comparisons may be made with *ITS2* greatly exceeds that of *psbA* ^ncr^ (*psbA* ^ncr^ sequences from the C3 radiation and the C1 radiation are largely unalignable).

As our ability to resolve Symbiodiniaceae improves, taxonomic descriptions may need to be revisited and updated. The majority of Symbiodiniaceae taxa descriptions to date include information related to *ITS2* sequence characterization (for example, but not limited to, Hume et al., 2015; Jeong et al., 2014; LaJeunesse, 2017; Lajeunesse, Parkinson, & Reimer, 2012). In many cases, this information relates only to the most common *ITS2* sequence associated with the taxa (e.g. C1, A1). However, more recent descriptions contain information relating to additional *ITS2* sequence variants that are instrumental in identifying the taxa in question using the *ITS2* marker (e.g. Hume et al., 2015; Hume et al., 2016; LaJeunesse et al., 2014). Indeed, we see that *ITS2* sequences referred to in such descriptions (e.g. *Cladocopium thermophilum* : C3, C3‐gulf; *Durusdinium trenchii* [formerly *Symbiodinium trenchii*]: D1, D4) are correspondingly being identified as DIVs in the SymPortal framework. Just as these more recent descriptions have exploited improvements in scientific understanding and sequencing power to offer more fine‐scale resolution, the improved resolution of the SymPortal framework should also be incorporated into taxonomic descriptions where appropriate. Where a unique *ITS2* type profile is able to identify a discrete Symbiodiniaceae taxon, incorporating information relating to its characterizing DIVs into its description will increase the ease with which taxa may be identified. Of course, further subdivisions of Symbiodiniaceae taxa proposed by differentiated *ITS2* type profiles will need to be corroborated with additional supporting evidence. This may take the form of fine‐scale genetic markers such as the chloroplastic *psbA* ^ncr^ and microsatellite flanking sequences, biogeographical data such as specific host‐species fidelities or physical distributions, or correlations of genetic divisions to host or symbiont physiological measurements.

Aside from considering how taxonomic descriptions may be advanced, it is equally important to consider how the outputted resolutions of SymPortal may be evaluated in relation to previous analyses that used alternative methods of analysis in conjunction with the *ITS2* marker. By reporting all levels of resolution from the coarser to the finer scale, namely clade, most abundant sequence, putative species and *ITS2* type profile, the SymPortal output enables researchers to work at the most appropriate level for their investigation, while maintaining the ability to compare with previous studies. For example, many early analyses reported only to the clade (genus) level, which may be compared directly to SymPortal's ‘clade’ output (see Section 2.3.6 for further details). More recently, samples were often assigned to a ‘type,’ a somewhat ambiguous term, often related to the most abundant sequence, or sequences, found in a sample. Such findings will be comparable using the ‘Majority *ITS2* sequence’ output of SymPortal. Previously used OTU clustering approaches may also be compared: those approaches that cluster within samples may be approximated to identifying the most abundant sequences of samples (therefore comparable again to the ‘Majority *ITS2* sequence’ output), and those that cluster across samples may be compared by searching for OTU representative sequences amongst *ITS2* type profile DIVs. With time, the new Symbiodiniaceae systematics will also be fully integrated into the SymPortal outputs to ensure both forward and reverse comparability of studies analysed through the SymPortal framework.

While we have demonstrated SymPortal's ability to resolve between closely related Symbiodiniaceae taxa, it is also important to consider whether this level of resolution is biologically pertinent. In the analysed data set, we have demonstrated SymPortal's ability to differentiate subtaxa within *Cladocopium thermophilum* that associate with specific scleractinian coral hosts (Figure 3). Analyses conducted since the initial description of *C. thermophilum* have returned a larger diversity of chloroplastic *psbA* ^ncr^ marker sequences from genotypes belonging to this species, than were found in the initial description (Hume et al., 2016). This additional diversity has led to the hypothesis that the *C. thermophilum* taxon probably contains multiple subtaxa that may be species in their own right. With the incorporation of *ITS2* intragenomic sequence variants into analyses, delineation of specific geographical and host‐specific populations has now been made possible (Hume, D’Angelo, Burt et al., 2018; Smith, Ketchum et al., 2017b). In particular, the analyses conducted with SymPortal here allow for the identification of these host‐specific subtaxa by their specific combinations of DIVs. The fidelity of a given algal symbiont to a specific coral host can be viewed as being representative of a complex and complementary suite of physiological mechanisms that result in the specific host–symbiont association (D'Angelo et al., 2015; Davy, Allemand, & Weis, 2012; Ochsenkuhn, Rothig, D'Angelo, Wiedenmann, & Voolstra, 2017; Voolstra et al., 2009). As such it would appear that the resolution offered by SymPortal is probably biologically significant. Importantly, it should be noted that Symbiodiniaceae taxa to date have been classified at a coarser resolution than is used here, either with alternative markers (e.g. Picciani, de Lossio e Seiblitz, de Paiva, e Castro, & Zilberberg, 2016; Thomas, Kendrick, Kennington, Richards, & Stat, 2014) or with alternative means of analysis using the *ITS2* marker (Howells, Abrego, Meyer, Kirk, & Burt, 2016; Hume et al., 2013). Given the biologically meaningful finer scale resolutions presented through the use of the SymPortal framework, generalizations based on these coarser resolutions should be exercised with care, as exceptions to these generalizations may simply be unresolved, if derived from more traditional classification approaches (e.g. Symbiodiniaceae containing the C3 sequence as their majority sequence are generally considered to be thermally sensitive). Yet, *C. thermophilum*, the predominant algal symbiont harboured by corals surviving in the world's hottest sea, the Persian/Arabian Gulf, also has a most abundant sequence of C3 (Howells et al., 2016; Hume et al., 2013; Smith, Vaughan et al., 2017c). However, only by undertaking further fine‐scale analyses and correlating these results with associated metadata will we be able to better assess at what level phylogenetic resolutions correlate with meaningful biological divisions.

The central analytical principle that the SymPortal and DGGE‐based analyses are built on has some inherent limitations. The principle relies on identifying re‐occurring sets (found in multiple samples) of *ITS2* sequences with the understanding that the more commonly a set of sequences occurs, the more likely that set of sequences is representative of a single taxon (LaJeunesse, 2001, 2002). However, although rare, it is possible that Symbiodiniaceae taxa co‐occur in a sufficient number of samples that the *ITS2* sequences returned from several taxa may be documented as being representative of a single taxon. In the analyses that have already been completed, such ‘super types’ have been a very rare occurrence with only one detected case so far. In this study (unpublished), an *ITS2* type profile was generated by SymPortal that was co‐dominant for the C15 and C3 sequences. This *ITS2* type profile was easily diagnosed as a ‘super type’ due to its profile being a combination of two *ITS2* type profiles also identified in the same study (one C3 radiation *ITS2* type profile, and one C15 radiation *ITS2* type profile). In cases where these super types have been identified, mitigations to their specific instances have been incorporated into the SymPortal algorithms directly to prevent their propagation and re‐occurrence. In the future, however, a median joining network‐based approach will probably be used to assess whether co‐occurring sets of sequences may represent several taxa. Sequence collections will be assessed to see whether they form contiguous networks characteristic of a single taxon's profile, or whether multiple discrete radiations exist, probably characteristic of multiple taxa. Another limitation occurs when trying to quantify the abundances of closely related symbiont taxa (taxa that share the same most abundant *ITS2* sequence) found in the same sample. In such cases, where several taxa in a single sample have some of the same DIVs in common, it is extremely difficult to partition what proportions of each of the DIVs are representative of which taxa (as it would be for any other approach given the nature of the problem). Currently, SymPortal uses a conservative approach and does not aim to partition the DIVs, but instead assigns only one of the taxa in question to the sample (the *ITS2* type profile containing the greatest abundance of DIVs from the sample). However, given that the majority of coral samples typically harbour one dominant Symbiodiniaceae taxon, especially within a single genus, we anticipate this to be an issue restricted to a small number of cases.

Free‐living or environmental assemblages of Symbiodiniaceae may be structured very differently to those assemblages associated with an animal host. Outside of the host, Symbiodiniaceae populations are free from any host‐selective influence and might therefore be more evenly distributed. Therefore, it cannot be assumed that a single Symbiodiniaceae taxon will be prevalent in a sample from the environment, an assumption required for SymPortal to be able to successfully identify *ITS2* type profiles of putative taxa (Thornhill et al., 2017, and references therein). However, as mentioned previously, with continued use, SymPortal will accrue a catalogue of *ITS2* type profiles representative of identified putative Symbiodiniaceae taxa. This catalogue of defining *ITS2* type profiles may be applied to *ITS2* amplicon libraries from environmental samples to predict the presence of Symbiodiniaceae that have already been identified in animal hosts. However, given the breakdown of the aforementioned assumption in these free‐living environments, it is unlikely to be straightforward to identify previously unaccounted putative taxa *de novo* from environmental samples. Notably, many of the free‐living taxa may not form stable associations with hosts (Thornhill et al., 2017). Besides attempting to use additional markers to attain a finer scale resolution in free‐living samples, another option would be to extract, culture, sequence and characterize monoclonal strains found in such samples (*sensu* Arif et al., 2014). While host‐associating Symbiodiniaceae can be notoriously difficult to culture, free‐living Symbiodiniaceae are often more readily cultured (Krueger & Gates, 2012; LaJeunesse, 2002; Santos, Taylor, & Coffroth, 2001). Once cultured, monoclonal lines may be sequenced and their *ITS2* type profiles directly submitted to the SymPortal database to enable their identification in subsequent analyses.

Basin‐ and global‐scale coral reef ecosystem sampling efforts (e.g. the *Tara* Oceans or *Tara* Pacific expeditions https://oceans.taraexpeditions.org/en/, or the Global Coral Microbiome Project http://coralmicrobes.org/) are bringing us closer to having a complete catalog of Symbiodiniaceae diversity (Decelle et al., 2018). Thanks to the wide use of the *ITS2* marker in many previously conducted analyses we can already observe closely related Symbiodiniaceae taxa (having the same most abundant *ITS2* sequence in common) harboured in a range of species found across geographical ranges that span ocean basins (e.g. Pettay, Wham, Smith, Iglesias‐Prieto, & LaJeunesse, 2015). As sampling efforts intensify, and sequencing and informatic power becomes more accessible, we may be able to document and catalogue such a global convergence of Symbiodiniaceae taxa but at an even finer scale, unlocking the biological inferences associated with this increased resolution. However, standardization between future sampling initiatives (i.e. use of methodologies that incorporate comparable approaches to resolution and nomenclature) will be critical in achieving this goal. To this end, SymPortal enforces standardization through consistent quality control parameters built‐in to the analytical framework and encourages integration and data curation through the offered ability to run SymPortal against the centralized SymPortal database via SymPortal.org. SymPortal is also a community‐driven platform, in that its power to resolve grows with the addition of more sequence data. By embracing this standardized and user‐driven approach, we hope that SymPortal will be widely adopted by the research community as a tool that can generate better results from data that are already being produced. We consider that SymPortal should play an instrumental role in making future sampling efforts comparable and in maximizing their efficacy in working towards the classification of the global Symbiodiniaceae diversity.

## CONFLICT OF INTEREST

The authors declare no conflicts of interest.

## AUTHOR CONTRIBUTIONS

The SymPortal framework was conceived by B.C.C.H., C.R.V., J.W., E.G.S. and M.Z. The framework was built by B.C.C.H. with significant input from: C.R.V. and J.W. for funding and provision of computational resources; C.R.V., E.G.S., M.Z. and T.L.J. for developing analysis theory; E.G.S. and J.B. for providing example data sets; H.J.M.W. for informatic support; and C.R.V. for sourcing additional data to aid in development of the framework prior to public release. B.C.C.H. conducted data analyses and created the figures with significant input from C.R.V., M.Z. and E.G.S. B.C.C.H. and C.R.V. wrote the manuscript; all authors commented and approved the final manuscript.

## Data Availability

Data from the original Smith, Vaughan et al. (2017c) study are deposited in the Dryad Digital Repository (http://dx.doi.org/10.5061/dryad.h6s54). The data used to create Figure 3 are available through the SymPortal wiki hosted on GitHub: https://github.com/didillysquat/SymPortal_framework/wiki. The largely programmatic process used to make figures 2–5 is documented here: https://github.com/didillysquat/sp_ms_figure_creation. Version 0.2.2 of the SymPortal framework's source code has been archived at https://doi.org/10.5281/zenodo.2552178.
