# Supplementary material for: SymPortal: A novel analytical framework and platform for coral algal symbiont next‐generation sequencing ITS2 profiling
Source: Mol Ecol Resour. 2019 Apr 26;19(4):1063–80. doi: 10.1111/1755-0998.13004 (PMC6618109; doi:10.1111/1755-0998.13004)
Supplement: Supplementary file 1 [file MEN-19-1063-s001.pdf]

SymPortal: a novel analytical framework and platform for coral algal symbiont next-generation sequencing *ITS2* profiling

Running title: SymPortal: coral algal symbiont NGS ITS2 profiling

Supplemental Information

Hume B. C. C.[1†], Smith E. G.[2], Ziegler M.[1], Warrington H. J. M.[3], Burt J. A.[2], LaJeunesse T. C.[4], Wiedenmann J.[5,6] & Voolstra C. R.[1†]

1 - Red Sea Research Center, Division of Biological and Environmental Science and Engineering (BESE), King Abdullah University of Science and Technology (KAUST), Thuwal, Saudi Arabia

2 - Center for Genomics and Systems Biology, New York University Abu Dhabi, Abu Dhabi, UAE

3 - Faculty of Computer Science and Technology, University of Cambridge, UK

4 - Department of Biology, The Pennsylvania State University, PA, USA

5 - Coral Reef Laboratory, Ocean and Earth Sciences, University of Southampton, Southampton, UK

6 - Institute for Life Sciences, University of Southampton, Southampton, UK

†Corresponding authors: Hume B. C. C., benjamin.hume@kaust.edu.sa; Voolstra C. R., christian.voolstra@kaust.edu.sa

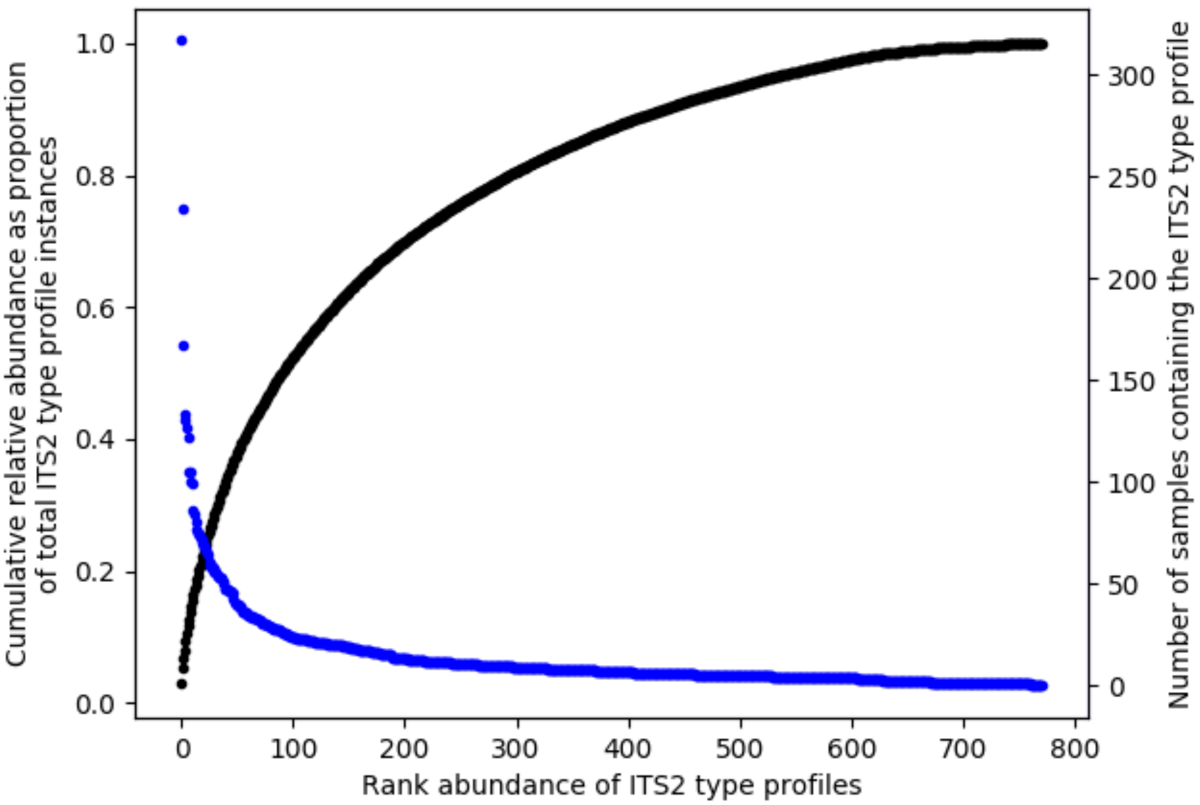

Supplemental Figure S1 – Abundance of the 771 *ITS2* type profiles in the latest SymPortal analysis (DataAnalysis UID 44). *ITS2* type profiles are ordered according to the number of samples that they are found in (‘Rank abundance of *ITS2* type profiles’). The number of samples each of the *ITS2* type profiles is found in is plotted in both absolute terms (‘Number of samples containing the *ITS2* type profiles’) and as a cumulative fraction of the total instances of *ITS2* type profiles, i.e. total number of *ITS2* type profile / sample association (Cumulative relative abundance as proportion of total *ITS2* type profile instances).

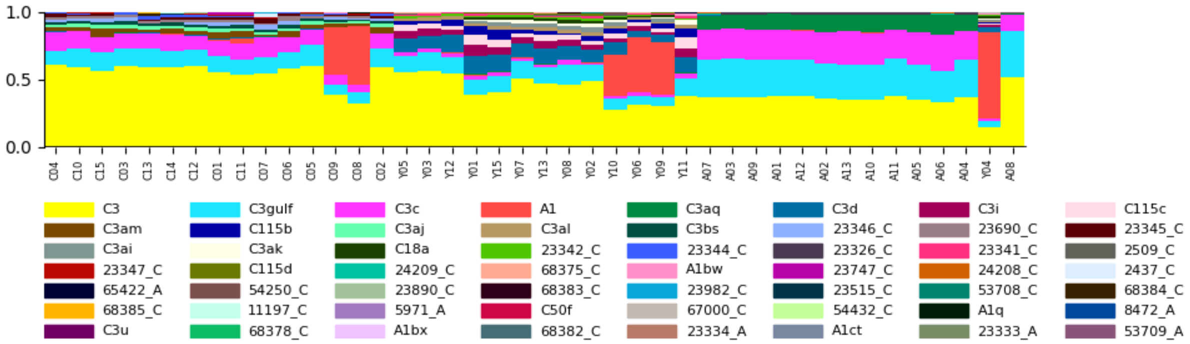

Supplemental Figure S2 – Relative abundance of post-quality control (post-QC) sequences across samples. This plot was automatically generated by SymPortal when conducting the analysis for this study. This figure has not been modified since its generation. Such a plot is automatically generated for every SymPortal data submission and data analysis to give an overview of the data. Each column represents data for a sample, where the different colours represent different relative abundances of post-QC ITS2 Symbiodiniaceae sequences. When output during data submission, samples are ordered according to similarity of the sequences they contain. When output during data analysis, samples are ordered according to similarity based on the ITS2 type profiles they contain.

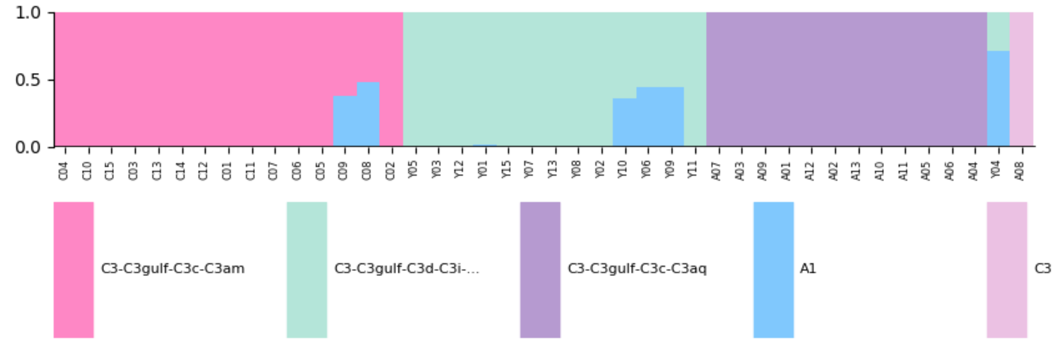

Supplemental Figure S3 – Relative abundance of predicted ITS2 type profiles across samples. This plot was automatically generated by SymPortal when conducting the analysis for this study. The figure has not been modified since its generation. The plot suggests specificity of ITS2 type profiles for each host genus, and hence, specific host-symbiont associations (at a resolution previously unattainable). Such a plot is automatically generated for every SymPortal data analysis to give an overview of the data. Each column represents data for a sample, where the different colours represent different relative abundances of predicted ITS2 type profiles found in the samples. Samples are ordered according to their abundance and similarity of predicted ITS2 type profiles.

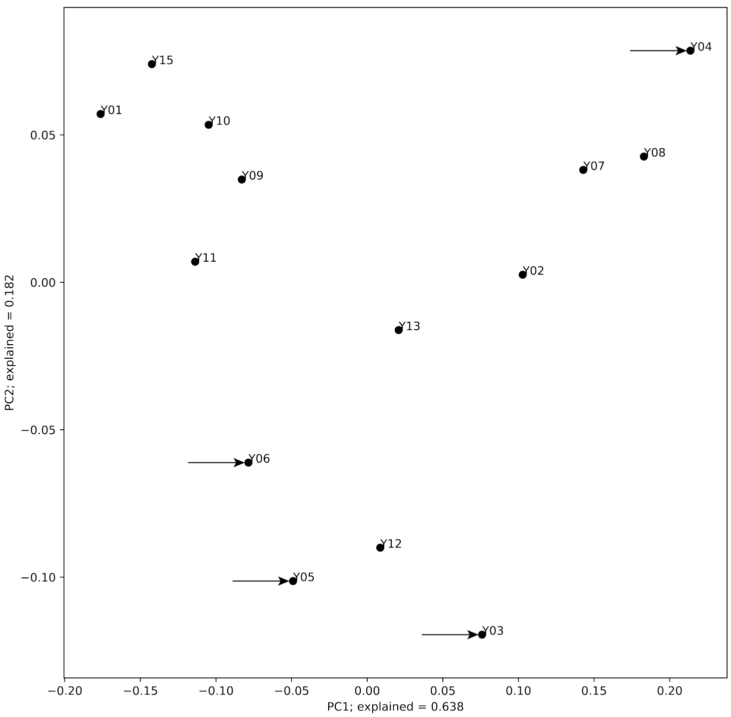

Supplemental Figure S4 –First two components of a Principal Coordinate Analysis plot of UniFrac-based distances between all *Platygyra* spp. samples based on post-QC ITS2 Symbiodiniaceae sequences. Samples in which parallelly determined *psbA*^{ncr} sequences differed from the *Platygyra* consensus sequence are annotated with an arrow. The percentage of difference explained by each of the components is annotated on the axes respectively.

Supplemental file legends

Supplemental File S1 – Tab delimited *ITS2* sequence output count table as absolute counts for the Smith et al 2018 dataset analysed through the SymPortal framework.

Supplemental File S2 – Tab delimited *ITS2* sequence output count table as relative proportion of total Symbiodiniaceae sequences for the Smith et al 2018 dataset analysed through the SymPortal framework.

Supplemental File S3 – .fasta file containing nucleotide sequence information for each *ITS2* sequence output in Supplemental File 1 and Supplemental File 2.

Supplemental File S4 – Tab delimited *ITS2* type profile output count table as absolute counts for the Smith et al 2018 dataset analysed through the SymPortal framework.

Supplemental File S5 – Tab delimited *ITS2* type profile output count table as relative proportion of total Symbiodiniaceae sequences for the Smith et al 2018 dataset analysed through the SymPortal framework.

Supplemental File S6 – UniFrac pairwise distances of *Cladocopium ITS2* type profiles identified for the Smith et al 2018 dataset analysed through the SymPortal framework.

Supplemental File S7 – Principal coordinate analyses coordinates of the UniFrac pairwise distances from Supplemental File 6.

Supplemental File S8 – UniFrac pairwise distances of samples considering only *Symbiodinium* (formerly clade A) sequences for the Smith et al 2018 dataset analysed through the SymPortal framework.

Supplemental File S9 – Principal coordinate analyses coordinates of the UniFrac pairwise distances from Supplemental File S8.

Supplemental File S10 – UniFrac pairwise distances of samples considering only *Cladocopium* sequences for the Smith et al 2018 dataset analysed through the SymPortal framework.

Supplemental File S11 – Principal coordinate analyses coordinates of the UniFrac pairwise distances from Supplemental File S10.

Supplemental File S12 – The .fasta file used to make the blastn database that was used to screen for clade C sequences.

Supplemental File S13 – The .fasta file containing the $psbA^{ncr}$ consensus sequences used to make the $psbA^{ncr}$ maximum likelihood phylogeny.
